# Temporal and Regional Variability in the Skin Microbiome of Humpback Whales along the Western Antarctic Peninsula

**DOI:** 10.1128/AEM.02574-17

**Published:** 2018-02-14

**Authors:** K. C. Bierlich, Carolyn Miller, Emelia DeForce, Ari S. Friedlaender, David W. Johnston, Amy Apprill

**Affiliations:** a Marine Chemistry and Geochemistry Department, Woods Hole Oceanographic Institution, Woods Hole, Massachusetts, USA; b Division of Marine Science and Conservation, Nicholas School of the Environment, Duke University Marine Laboratory, Beaufort, North Carolina, USA; c Marine Mammal Institute, Oregon State University, Newport, Oregon, USA; d Institute of Marine Sciences, Department of Ecology and Evolutionary Biology, University of California Santa Cruz, Santa Cruz, California, USA; INRS—Institut Armand-Frappier

**Keywords:** Antarctica, SSU rRNA gene, bacteria, humpback whale, skin, temporal

## Abstract

The skin is the first line of defense between an animal and its environment, and disruptions in skin-associated microorganisms can be linked to an animal's health and nutritional state. To better understand the skin microbiome of large whales, high-throughput sequencing of partial small subunit rRNA genes was used to study the skin-associated bacteria of 89 seemingly healthy humpback whales (Megaptera novaeangliae) sampled along the Western Antarctic Peninsula (WAP) during early (2010) and late (2013) austral summers. Six core groups of bacteria were present in 93% or more of all humpback skin samples. A shift was observed in the average relative abundances of these core bacteria over time, with the emergence of four additional core groups of bacteria that corresponded to a decrease in water temperature, possibly caused by season- or foraging-related changes in skin biochemistry that influenced microbial growth, or other temporal factors. The skin microbiome differed between whales sampled at several regional locations along the WAP, suggesting that environmental factors or population may also influence the whale skin microbiome. Overall, the skin microbiome of humpback whales appears to provide insight into animal- and environment-related factors and may serve as a useful indicator for animal health or ecosystem alterations.

**IMPORTANCE** The microbiomes of wild animals are currently understudied but may provide information about animal health and/or animal-environment interactions. In the largest sampling of any marine mammal microbiome, this study demonstrates conservation in the skin microbiome of 89 seemingly healthy humpback whales sampled in the Western Antarctic Peninsula, with shifts in the bacterial community composition related to temporal and regional variability. This study is important because it suggests that the skin microbiome of humpback whales could provide insight into animal nutritional or seasonal/environment-related factors, which are becoming increasingly important to recognize due to unprecedented rates of climate change and anthropogenic impact on ocean ecosystems.

## INTRODUCTION

As long-living mobile mesopredators that require vast quantities of food for individual and population growth, large whales can serve as indicators of the health of marine environments (1). Rapid environmental change has been shown to significantly influence the health and likelihood of disease in marine organisms (2); thus, our ability to monitor the health and status of large whale populations is growing in importance. While health indices derived from photogrammetry of body condition (3, 4) and reproductive and stress hormones (5) exist, there is still a need to develop other integrative health assessment approaches to provide additional context for the immediate health state of these animals and their risk of infection or disease.

Recent evidence indicates that communities of microorganisms (microbiomes) associated with animals, including humans, provide an indication of health, as well as other individual traits (6, 7). For example, mice with skin disorders possess an altered abundance of the main or core bacterial members of their skin microbiome, suggesting a connection between skin health and the microbiome (8). Recently, there has been an increase in the number and types of skin lesions reported on cetaceans (9). In fact, numerous viruses, pathogenic bacteria, and fungi were associated with skin lesions from immunocompromised cetaceans and from cetaceans with higher exposure to pollution (9). Additionally, skin lesions in the endangered North Atlantic right whales (Eubalaena glacialis) correlated with a longer interval between calving, suggesting a connection between nutritional and/or hormonal health and skin condition (10). The microbiomes of marine mammals are underexplored, but recent research suggests the existence of species-specific assemblages (11, 12). In some populations of humpback whales (Megaptera novaeangliae), the skin was found to contain a core group of bacteria made up of Tenacibaculum and Psychrobacter spp., and shifts in this core group, as well as the presence of potential pathogens, were correlated with entangled and stranded whales (13). While deciphering whether the microbiome affects the probability of infection or infection affects the microbiome is difficult in any animal system, it is particularly challenging for large whales because of their relative inaccessibility. Thus, understanding the composition of the skin microbiome in healthy large whales and the factors contributing to variability are the first steps that will lead to a better understanding of the relationship between the skin microbiome, animal health, and potentially, ecosystem health.

Humpback whales are a particularly interesting species for microbiome studies because they are found in every ocean and regularly migrate between high-latitude summer feeding grounds and low-latitude winter breeding grounds, thus exposing their skin to a wide variety of oceanic environments (14, 15). A recent study of humpback whale skin found that certain bacteria were common among whales from the North Pacific, South Pacific, and North Atlantic oceans (13). The study also detected differences in the bacterial communities between whales in feeding and breeding areas (feeding generally absent on the breeding areas), suggesting that metabolic activity (anabolism versus catabolism) and/or the rate of skin turnover from the different water temperatures are factors influencing the skin microbiome (13).

The Western Antarctic Peninsula (WAP) is an important foraging area for migrating humpback whales during the austral summer (16). While Southern Hemisphere humpback whales are recovering from overexploitation by commercial whaling during the 20th century (17), climate change along the WAP may pose a new threat to this recovery, as winter air temperatures have increased by over 5°C, and seasonal sea ice duration has decreased by over 80 days within the past 50 years (18–20). These changes could have considerable consequences on the Antarctic food web, since the replenishment of krill, the major food source for many marine predators, such as whales, seals, and seabirds, is dependent on the extent and duration of winter sea ice (19, 21). Although humpback whales are known to range broadly during the austral feeding season (22, 23), their distribution changes throughout that season (24): they move to a more coastal, dense, and restricted distribution following the seasonal movement of their prey (25, 26).

To further elucidate the relationship between foraging and spatial and temporal variations in the skin microbiome, we examined the skin microbiomes of humpback whales sampled along the WAP (see Fig. 7) at two time points corresponding to differences in their feeding season, early summer, when the animals first arrive at the feeding grounds, and late fall, after a full season of foraging, using small subunit rRNA (SSU rRNA) gene sequencing-based methods. Skin samples (see Table 4) were collected only from animals that were deemed to be in normal or robust health, which was determined by a visual assessment of body condition before each sample collection. Our results indicate that the composition of the skin microbiome changes temporally, and that the early foraging season of 2013 was different from that of the late season of 2010. Differences in the skin microbiomes were also observed between whales inhabiting different geographic regions along the WAP.

## RESULTS

### Core skin microbiome reflects seasonal shifts.

**FIG 1:**
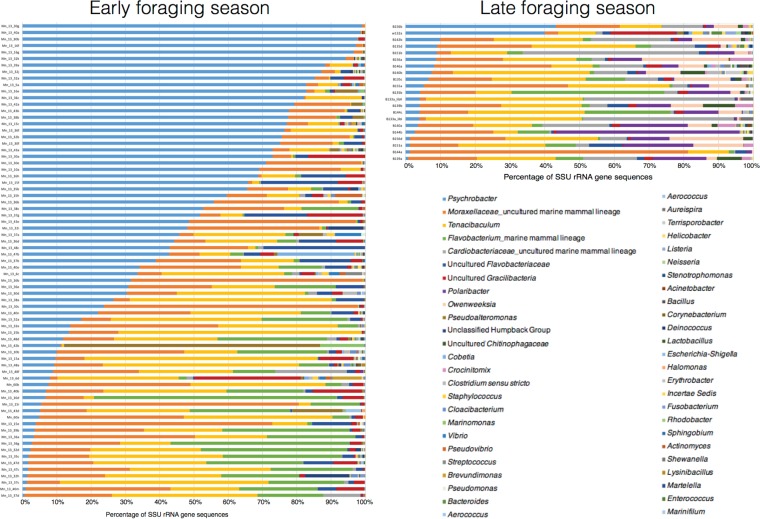
Relative abundance and taxonomic composition of the humpback whale skin microbial communities based on bacterial SSU rRNA gene sequences classified to the most resolving taxonomic level, either family, genus, or unclassified group name.

An average of 81,558 SSU rRNA gene sequences were examined per skin sample, with a range of 9,436 to 1,898,477 sequences. Across all 94 skin samples from 89 humpback whales, 49 groups of bacteria (generally genera) were identified (Fig. 1), and no sequences classified as archaea. Thus, the term “microbiome” here refers to bacteria. Psychrobacter, Tenacibaculum, and an uncultured group from the Moraxellaceae family that was previously identified in dolphin blowholes and mouths (Tursiops truncatus) (GenBank accession no. FJ959923 and KC260401) (11, 27) and humpback whale skin (GenBank accession no. GU201992) (28) (here referred to as Moraxellaceae uncultured marine mammal-associated lineage) were present in all samples (Fig. 1). To identify the core bacteria associated with the humpback skin, two analyses were conducted (explained in Materials and Methods). First, bacteria (represented by a number of distinct MED nodes) present in >93% of skin samples were considered the “core” microbiome. Second, bacteria present in 50 to 92% of the skin samples were considered the “common” microbiome. Based on these criteria, skin from early foraging season contained six core genera: Psychrobacter, Tenacibaculum, an uncultured Moraxellaceae lineage reported previously from marine mammals, a lineage of Flavobacterium reported previously from marine mammals, an uncultured Flavobacteriaceae organism, and an uncultured Gracilibacteria sp. (Table 1). Samples from late foraging season also had these same six core bacteria, as well as an additional four core groups of bacteria: Polaribacter, Owenweeksia, Crocinitomix, and an uncultured lineage from the Cardiobacteriaceae family (most similar to GenBank accession no. GU202000 from humpback whale skin [28] and GenBank accession no. FJ959944 from dolphin blowholes [27]) (Table 1). All of these core microbiome members, except Crocinitomix, were present in all of the late foraging season skin samples (Table 1).

**TABLE 1:** Description of the core microbiome members identified in humpback whale skin occurring along the WAP during early summer 2013 and late fall 2010^*a*^

Core group	Proportion found within no. of samples (%)	No. of MED nodes in each group	Name(s) of MED nodes
Core for early 2013 samples			
Psychrobacter	73/73 (100)	67	3568, 3845, 3239, 3470, 2464, 2405, 550, 43, 2473, 3575, 2470, 3735, 3759, 3569, 546, 3574, 3496, 2466, 2411, 3758, 3571, 1679, 3498, 788, 1394, 3244, 3501, 558, 3736, 2477, 3814, 2472, 3811, 559, 3217, 561, 555, 1678, 790, 3813, 32, 3494, 3714, 549, 3715, 3846, 2476, 3240, 2404, 560, 3812, 3572, 81, 2468, 39, 3847, 3547, 3570, 3761, 3471, 2469, 2412, 3219, 3576, 3243, 3549, 3502
Moraxellaceae uncultured marine mammal-associated lineage	73/73 (100)	26	3642, 3638, 2844, 3056, 3617, 2993, 3000, 1093, 2892, 2960, 2896, 3001, 1753, 3643, 3649, 3645, 3639, 2850, 3648, 1112, 1103, 3003, 3616, 10, 2893, 2998
Tenacibaculum	73/73 (100)	28	3612, 3415, 3376, 2238, 441, 2196, 3785, 2197, 14, 2293, 2594, 3421, 2595, 2240, 3379, 3419, 448, 123, 3424, 633, 2294, 3789, 2541, 3790, 3416, 2540, 2200, 3380
Flavobacterium marine mammal-associated lineage	72/73 (98.6)	9	2058, 2110, 2062, 261, 2111, 2061, 2064, 262, 1197
Uncultured Flavobacteriaceae	70/73 (95.9)	7	3337, 257, 2158, 3341, 451, 3343, 2155
Uncultured Gracilibacteria	68/73 (93.2)	9	630, 634, 2641, 629, 637, 2643, 641, 2640, 2644
Core for late 2010 samples			
Psychrobacter	21/21 (100)	67	3568, 3845, 3239, 3470, 2464, 2405, 550, 43, 2473, 3575, 2470, 3735, 3759, 3569, 546, 3574, 3496, 2466, 2411, 3758, 3571, 1679, 3498, 788, 1394, 3244, 3501, 558, 3736, 2477, 3814, 2472, 3811, 559, 3217, 561, 555, 1678, 790, 3813, 32, 3494, 3714, 549, 3715, 3846, 2476, 3240, 2404, 560, 3812, 3572, 81, 2468, 39, 3847, 3547, 3570, 3761, 3471, 2469, 2412, 3219, 3576, 3243, 3549, 3502
Moraxellaceae uncultured marine mammal-associated lineage	21/21 (100)	26	3642, 3638, 2844, 3056, 3617, 2993, 3000, 1093, 2892, 2960, 2896, 3001, 1753, 3643, 3649, 3645, 3639, 2850, 3648, 1112, 1103, 3003, 3616, 10, 2893, 2998
Tenacibaculum	21/21 (100)	28	3612, 3415, 3376, 2238, 441, 2196, 3785, 2197, 14, 2293, 2594, 3421, 2595, 2240, 3379, 3419, 448, 123, 3424, 633, 2294, 3789, 2541, 3790, 3416, 2540, 2200, 3380
Flavobacterium marine mammal-associated lineage	21/21 (100)	9	2058, 2110, 2062, 261, 2111, 2061, 2064, 262, 1197
Uncultured Flavobacteriaceae	21/21 (100)	7	3337, 257, 2158, 3341, 451, 3343, 2155
Uncultured Gracilibacteria	21/21 (100)	9	630, 634, 2641, 629, 637, 2643, 641, 2640, 2644
Cardiobacteriaceae uncultured marine mammal-associated lineage	21/21 (100)	8	3678, 817, 3059, 1094, 3058, 3065, 3061, 3687
Polaribacter	21/21 (100)	1	2352
Owenweeksia	21/21 (100)	1	1192
Crocinitomix	20/21 (95.2)	2	3308, 3309

a Bacterial groups were identified using minimum entropy decomposition (33) of bacterial SSU rRNA gene sequences.

**FIG 2:**
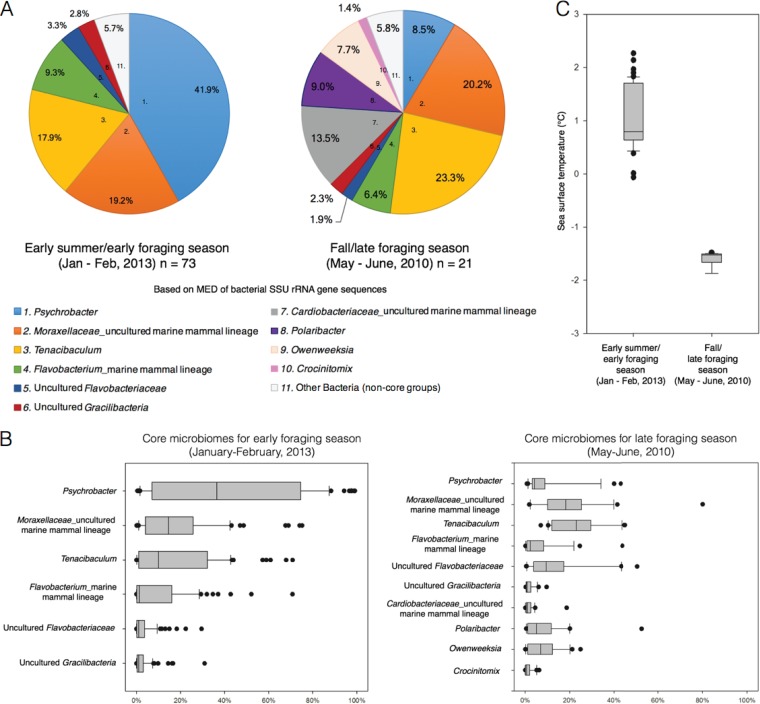
Distributions of the core microbiomes by foraging seasons, based on minimum entropy decomposition (MED) (29) of bacterial SSU rRNA gene sequences. (A) Pie charts representing the average abundance of each core microbiome lineage for both early and late foraging season. (B) Boxplot distributions, with average and standard deviation of the relative abundance of each core microbiome group for both early (left) and late (right) foraging season. (C) Boxplot distributions of moderate-resolution imaging spectroradiometer (MODIS)-Aqua SST (in degrees Celsius) at monthly average 9-km spatial scale for each GPS location of sample collection.

**FIG 3:**
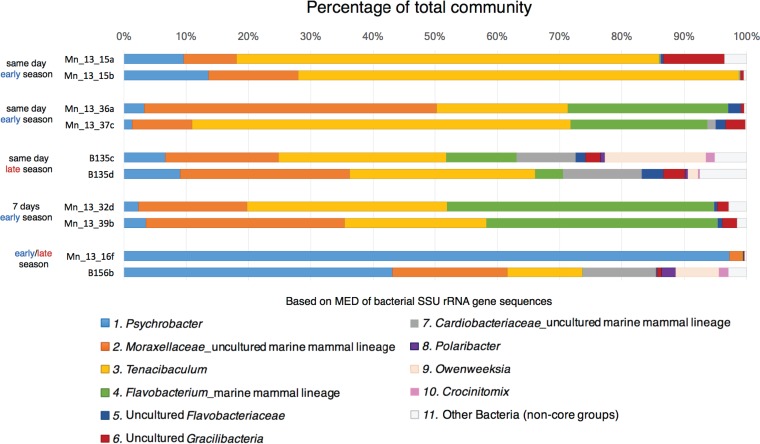
Relative abundance of the core bacteria in humpback whale skin samples from five individual whales that were resampled across the same or different season. Data are based on minimum entropy decomposition (MED) (29) of bacterial SSU rRNA gene sequences.

Psychrobacter spp. had the greatest change in relative abundance on the skin between seasons compared to the other six core bacterial groups present in both seasons. Specifically, Psychrobacter spp. decreased from an average relative abundance of 41.9% in early foraging season to 8.5% in late foraging season (Fig. 2A and B). The decrease in relative abundance of Psychrobacter spp. was also evident in the resampled individual whose skin was sampled during both seasons: 97.3% (early season) to 43.2% (late season) (Fig. 3; sample identification numbers [IDs] Mn_13_16f early and B156b late). The other resampled individuals showed similar skin microbiomes over the day to weeks of sampling (Fig. 3). An analysis of variance showed that the seasonal change in the relative abundance of Psychrobacter corresponded to a decrease in sea surface temperature (SST) from early to late foraging season [*F*(1, 115) = 519.3, *P* < 0.001] (Fig. 2C).

The common bacteria (present in 50 to 92% of samples) for early foraging season included Staphylococcus, Pseudomonas, an uncultured lineage from the Cardiobacteriaceae family previously reported in marine mammals, Pseudoalteromonas, Streptococcus, Cobetia, and Clostridium sensu stricto (see Table S1 in the supplemental material). These bacteria were present at fairly low average abundances (<1.7% of the community) (Fig. S1). In the late foraging season, the common bacteria included Pseudomonas, Staphylococcus, uncultured Chitinophagaceae, Clostridium sensu stricto, Cloacibacterium, Streptococcus, Corynebacterium, and uncultured Chitinophagaceae sp. and were also present at low average relative abundances (up to 1.6%) (Fig. S1).

### Microbial diversity increases during foraging season.

**FIG 4:**
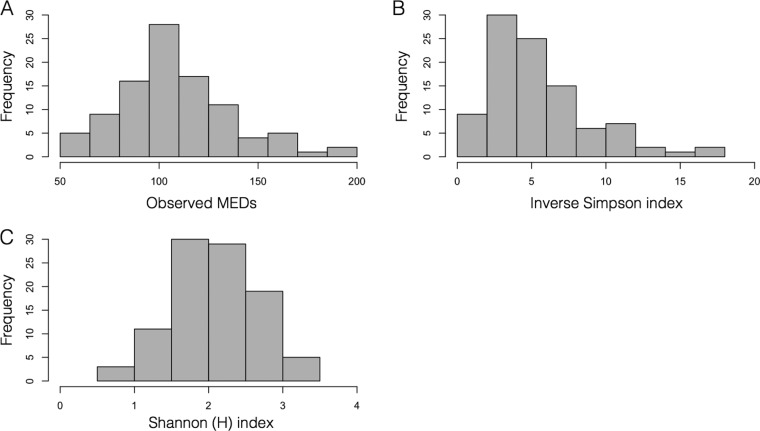
Histograms displaying the number of observed minimum entropy decomposition nodes (MED nodes) (29) of bacterial SSU rRNA genes (A), and other indices calculated from the MED nodes, including inverse Simpson index (B) and Shannon index, H (C).

Minimum entropy decomposition (MED) (29) was used to cluster the sequences, and the number of MED nodes identified in the skin samples ranged from 58 to 187 per sample, with an average of 108.3 nodes (Fig. 4A). The average inverse Simpson index was 5.4, and the Shannon index was 2.1, suggesting that the majority of skin samples contained microbiomes of similar diversity (Fig. 4). No differences in species richness and diversity between skin microbiomes of whales from different geographic regions were found (Table 2). However, the late-season samples were significantly more diverse than the early season samples (Table 2). No differences were detected in observed MED nodes between the seasons (Table 2).

**TABLE 2:** ANOVA results showing differences in the alpha-diversity measures as determined by SSU rRNA gene sequencing of the microbiome associated with humpback whales residing in different locations along the Western Antarctic Peninsula and time points

Alpha-diversity measure	ANOVA measure			
	*df*	Sum of squares	*F* value	*P* value
Location				
Observed MED nodes	8	7,970	1.467	0.182
Inverse Simpson index	8	108.8	1.63	0.129
Shannon (H) index	8	3.663	1.585	0.142
Time point (foraging season)				
Observed MED nodes	1	165	0.23	0.633
Inverse Simpson index	1	254.3	32.56	0.001^*a*^
Shannon (H) index	1	6.21	23.99	0.001^*a*^

a *P* ≤ 0.001

### Influences of foraging season, sex, and location on the composition of the skin microbiome.

**FIG 5:**
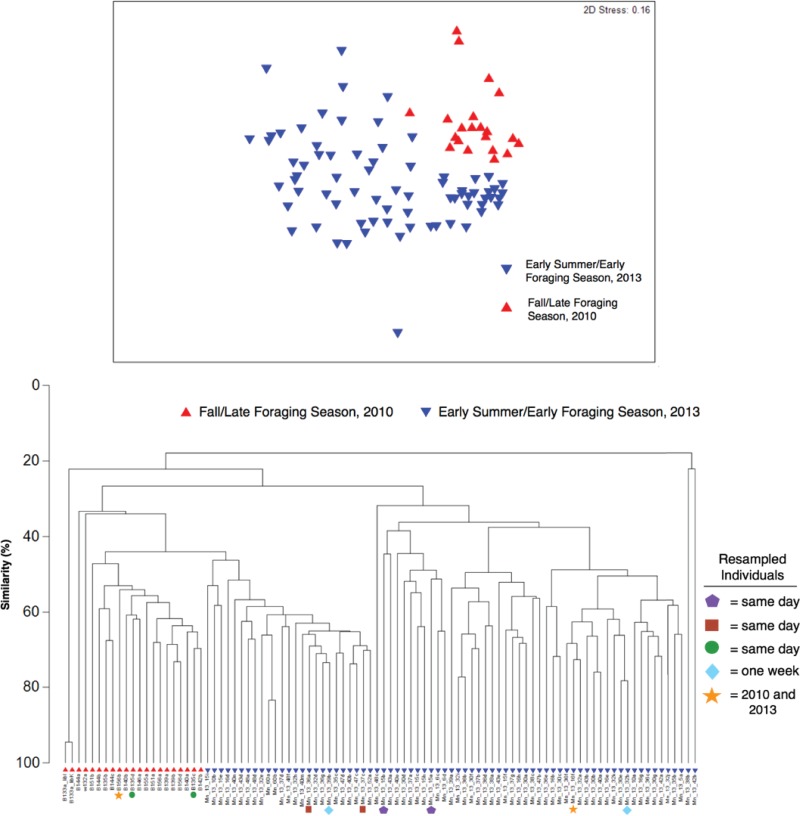
Nonmetric multidimensional scaling (NMDS) ordination analysis (top) and cluster dendrogram analysis (bottom) based on minimum entropy decomposition (MED) (29) of bacterial SSU rRNA gene sequences from humpback whale skin. Resampled individuals are represented by the symbols in the legend.

A nonmetric multidimensional scaling (NMDS) analysis and dendrogram clustering analysis of SSU rRNA genes from bacteria compared using the Bray-Curtis similarity index demonstrated that humpback skin contained bacterial communities that were different between the early and late foraging seasons (Fig. 5). A permutational multivariate analysis of variance (PERMANOVA) confirmed that the beta diversity, measured by the skin microbial community composition, differed significantly between early and late foraging seasons (Table 3).

**TABLE 3:** Results of PERMANOVAs examining the influence of sex, location, and time point on the humpback skin microbiome

Variable	*df*	Sum of squares	Pseudo-*F* value	*t* value	*P* value
Sex	2	5,970	1.34		0.159
Location	7	32,415	2.27		0.001^*a*^
Time point (foraging season)				3.61	0.001^*a*^

a *P* ≤ 0.001.

**FIG 6:**
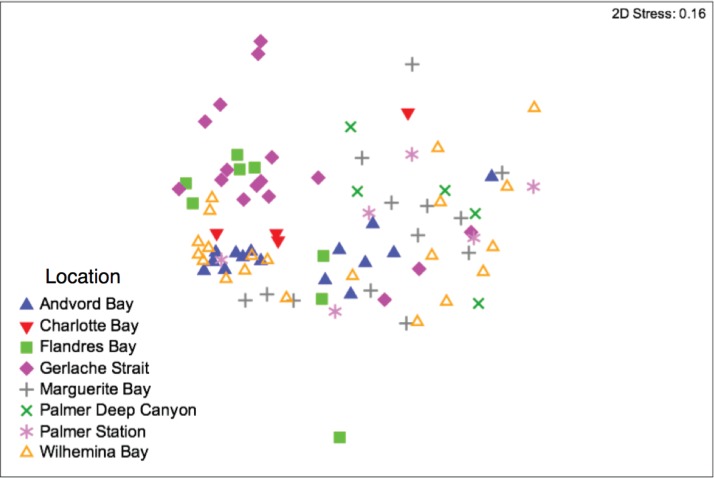
NMDS analysis of the skin microbiome of humpback whales collected from different locations along the Western Antarctic Peninsula, based on minimum entropy decomposition (MED) (29) of bacterial SSU rRNA gene sequences from humpback whale skin.

The influence of sex on the skin bacteria was also examined, and an NMDS ordination did not indicate any sex-related clustering (Fig. S2), nor did PERMANOVA specify any significant differences in the skin-associated bacteria that were related to sex (Table 3). Some clustering of bacterial communities by regional geographic area was found using a NMDS ordination comparing the eight locations containing ≥4 skin samples, and a PERMANOVA confirmed that whales sampled in these locations had significantly distinct skin-associated bacteria (Fig. 6 and Table 3). These differences were further elucidated in pairwise PERMANOVA comparisons, which indicated that skin microbiomes from the humpbacks sampled in Gerlache Strait were significantly different from those at the other sites, while samples from Wilhelmina Bay were the least different from those at the other locations (Table S2).

## DISCUSSION

The present study provides new evidence for a conserved core microbiome residing on the skin of humpback whales in the Southern Ocean. This study also provides insights into variations in the skin microbiome of humpbacks that are potentially related to foraging season and/or year, as well as regional geography.

Defining core microorganisms in microbiome studies can be done with a variety of approaches, which generally are defined by the study goals (30, 31). Using a fine-resolution index for operational taxonomic units (OTUs) (MED nodes), we identified several closely related MED nodes belonging to the same groups (generally genera) that comprised the core and common microbiome, and these core and common microbiome categories were defined by a natural division in our data. This approach appears to be ideal for this study because of the large number of closely related MED nodes identified for Psychrobacter (67 MED nodes) and Tenacibaculum (28 MED nodes). Currently, the sequence types within each of these genera are not known to be physiologically or ecologically distinct. While there is no direct knowledge about the genomes or physiology of Psychrobacter and Tenacibaculum spp. residing on humpback skin, species from these genera have been studied in other environments. Psychrobacter spp. are associated with many different marine organisms (32, 33) and are tolerant of extremely cold temperatures (33, 34). Tenacibaculum spp. are also generally cold-tolerant bacteria, previously found in diseased marine and freshwater fish (32, 35, 36). While it is possible that these cells may be pathogens to the whales, the animals appeared to be healthy, according to the field-based visual assessments of body condition. Tenacibaculum spp. can prey on other bacteria (37) and may play a beneficial role in the maintenance of the skin microbiome in whales. Other core bacteria associated with humpback skin do not have cultivated representatives or genomic information, but several have been previously identified in other marine mammals. Specifically, Moraxella spp. were cultured from bowhead whale skin lesions (38). However, the most closely related sequences were found to be associated with healthy humpback whale skin (28) and the blowholes and mouths of dolphins (11, 27). Overall, this evidence suggests that the Moraxella spp. identified here may specifically reside with cetacean hosts.

A number of factors could be related to the observed changes in the skin microbiome throughout the course of the foraging season. For one, bacterial growth is tightly regulated by the substrates available; as such, alterations in the biochemistry and pH of the skin that are linked to nutrition may have influenced the observed shift in the skin-associated microbiome. Specifically, the lipid composition of the epidermis may change during the feeding season, as seen in human diet-skin biochemistry studies (39), which could result in a microbial community shift. Characterization of the epidermal lipid and protein content throughout the season in the context of the microbiome is needed to understand this connection. Another possible factor controlling the skin microbiome is that biofilms of diatoms and other protistan epibionts can develop on the skin when cetaceans are residing in these foraging grounds (40), and the associated microbiome of these diatom and protistan epibionts could host the additional core bacteria that were detected later in the foraging season.

Temperature is an important growth factor for bacteria and may be another factor contributing to the skin microbiome composition of humpback whales. A decrease in SST was observed between the two time points studied, which may have caused differential growth in the bacteria. Also, colder water can cause cetaceans to slough skin at lower rates than warmer water. For example, beluga whales (Delphinapterus leucas) undergo a prompt migration into warm brackish waters where their skin undergoes much more rapid sloughing than when they are in their regular colder habitat (41). A quick migration also was observed in killer whales (Orcinus orca) from Antarctica to tropical waters and back, presumably for skin maintenance (40). Skin sloughing rates due to water temperature differences could affect the amount and kinds of organisms represented in the microbiome throughout the season; hence, the seasonal shift in the skin microbiome observed here may be reflecting the physiological constraint of lower skin sloughing and regeneration rates.

Overall, while there is clearly a shift in the core skin microbiome from early to late foraging season, it should be considered that the samples collected from these time frames are also from two different years, 2010 and 2013. It is possible that these observed shifts are driven by a yearly or other difference, such as variations in water temperature and food quality and quantity. It is important to note that samples collected from 2013 fall into two groups in the cluster dendrogram analysis, where approximately one-third of the skin microbiomes (25 of 73 samples) were more closely related to the microbiomes from 2010 (Fig. 5). The microbiome of one whale resampled within a week in 2013 appeared in both of these clusters (blue diamonds in Fig. 5). While weekly variability was evident, the microbiome of a whale resampled on the same day was less variable, falling within the same cluster (green circles, purple pentagons, and red squares in Fig. 5). Indeed, the decrease in relative abundance of Psychrobacter spp. was evident in the one whale whose skin was sampled during both seasons (and years) (Fig. 3). It is also interesting to note that this whale was sampled in Flandres Bay (2010) and Marguerite Bay (2013), two bays hosting whales that had skin microbiomes that were statistically distinct from each other (Table S2). Tracking the location and examining the skin microbiomes from the same individuals throughout a single foraging season would help clarify the findings presented here.

It is also important to note that seawater samples were not analyzed in this study. However, previous studies showed that the microbial community on humpback whale skin is significantly different from the community present in proximate seawater (13, 28); therefore, variation in the skin microbiome likely is not attributable to microbial alterations occurring in the surrounding water but should be included in future studies.

### Conclusions.

Identifying a core microbiome is the first step toward defining a healthy or normal microbial community and predicting the response of that community to any perturbation (31). With climate change altering the ocean environment at unprecedented rates, it is particularly important to understand how large whales and other keystone species and the ecosystems they inhabit are impacted. Adaptive management strategies that include long-term health and population monitoring and forecasting tools to understand how mobile vertebrates will be affected are still needed (2). As such, the skin microbiome of large whales is a potentially useful tool that could help assess the status of whale populations, including foraging success, as well as indicate changes in the ocean ecosystem.

## MATERIALS AND METHODS

### Skin samples.

**FIG 7:**
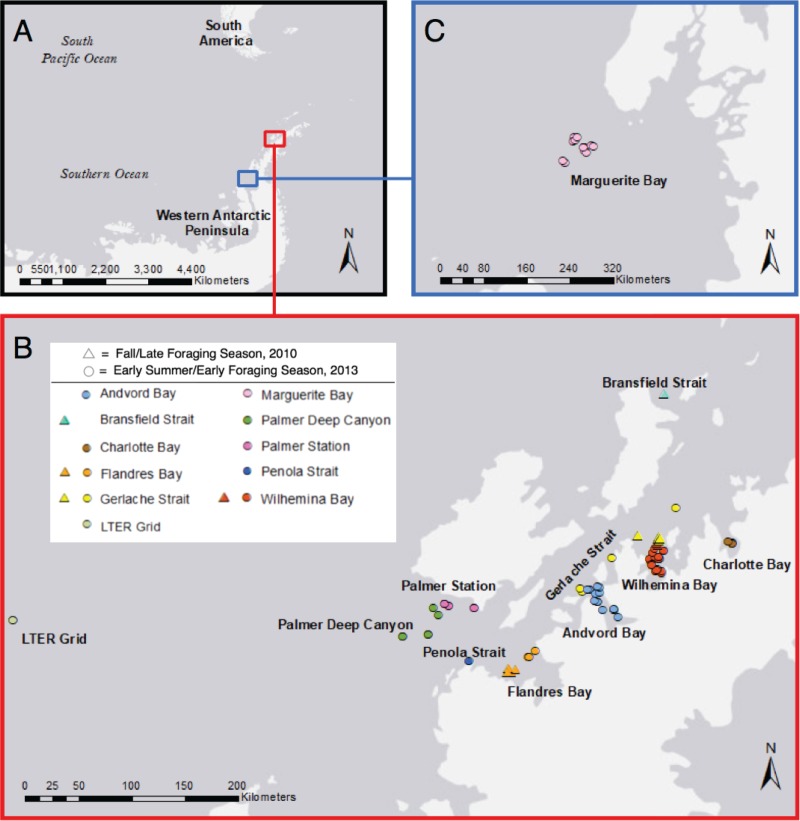
Map describing locations where humpback whale skin samples were collected along the Western Antarctic Peninsula (A), Western Antarctic Peninsula and Gerlache Strait (B), and Marguerite Bay (C). The maps were created with ArcMap GIS software (version 10.5.0; Esri, Inc.) using ArcMap's World Light Gray Base service layer. LTER, Palmer Long-Term Ecological Research.

Ninety-four skin samples were collected from 89 humpback whales in 12 different locations along the Western Antarctic Peninsula (WAP) during May and June 2010 (fall/late foraging season) and January and February 2013 (early summer/early foraging season) (Fig. 7 and Table 4). Five individuals were sampled twice during the study; three individuals were resampled on the same day, one a week later, and one was sampled in 2010 and 2013. Because the range of sample sizes at each of the locations within the WAP was varied (1 to 21), statistical tests comparing locations only focused on the eight WAP locations that had a sample size of ≥4 (Andvord Bay, *n* = 15; Charlotte Bay, *n* = 4; Flandres Bay, *n* = 8; Gerlache Strait, *n* = 16; Marguerite Bay, *n* = 13; Palmer Deep Canyon, *n* = 6; Palmer Station, *n* = 6; and Wilhemina Bay, *n* = 21) (see Table 4). Three locations contained samples from both 2010 and 2013 (Flandres Bay, Gerlache Strait, and Wilhemina Bay) (Fig. 7 and Table 4).

**TABLE 4:** Number of humpback whale skin samples collected by standard biopsy techniques and examined for each location along the WAP

Location along WAP	No. of samples collected				
	Total	Early summer/early foraging season (*n* = 73)		Fall/late foraging season (*n* = 21)	
		January 2013	February 2013	May 2010	June 2010
Andvord Bay	15		15		
Bransfield Strait	1			1	
Charlotte Bay	4		4		
Flandres Bay	8		3	1	4
Gerlache Strait	16		4	12	
LTER grid	2	2			
Marguerite Bay	13	13			
Palmer Deep Canyon	6	6			
Palmer Station	6	4	2		
Penola Strait	1		1		
Unknown	1			1	
Wilhemina Bay	21		19	2	
Total	94	25	48	17	4

Body condition was visually assessed in the field by scoring the whales as emaciated, normal, or robust. Indications of emaciation were: a concave shape around the spinal column with depressed axial musculature, a visible indentation at the base of the skull, or a visible outline of the scapula near the insertion of the flipper. Whales without these features were scored as normal, and whales that were convex along the spinal column were considered robust. All animals that were sampled as part of this study were deemed to be in robust or normal body condition; no emaciated animals were sampled.

Skin samples were collected from the upper flank near the dorsal fin of humpback whales via standard biopsy techniques. Specifically, a 68-kg pull crossbow and floating Ceta-Dart darts with 40-mm surgical stainless steel tips collected a small skin and blubber sample roughly 5 mm wide and 20 to 30 mm deep, depending on the angle of the sampling dart on impact (42, 43). Tips were sterilized using 8.25% concentrated bleach, allowed to dry, cleaned, and then doused in 100% ethyl alcohol before each use. The darts were retrieved by hand and the tips immediately placed into a sterile bag (such that the sample was not contaminated) and on ice for no more than 10 h before transfer to a cryovial via sterile tools for freezing at −80°C. The global positioning system (GPS) location and the regional names of the bay, strait, etc. were recorded for each skin sample collected. One skin sample, w132a, was collected from an acoustic tag placed on the animal.

### DNA extraction, amplification, and sequencing.

DNA was extracted from 1 to 30 mg of the top layer of the epidermis, with an average of 13 mg, from each sample using a DNeasy tissue kit (Qiagen, Valencia, CA, USA) and quantified using the Invitrogen Qubit 2.0 assay fluorometer (Life Technologies, Beverly, MA, USA). The V4 region of the SSU rRNA gene was amplified using barcoded primers (515F-Y, 5′-GTGYCAGCMGCCGCGGTAA-3′; and 806RB, 5′-GGACTACNVGGGTWTCTAAT-3′) (44, 45), broadly targeting bacteria and archaea. Samples and sterile water (negative control) were amplified in triplicate using PCR on a S1000 thermal cycler (Bio-Rad Laboratories, Hercules, CA, USA), as follows: 2 min at 95°C, followed by 30 to 35 cycles of 20 s at 95°C, 15 s at 55°C, and 5 min at 72°C, followed by 10 min at 72°C. Triplicate PCR mixtures contained 1 μl of DNA, 5 μl of GoTaq 5× Flexi buffer, 14.75 μl of H_2_O, 2.5 μl of a 25 nM MgCl_2_ solution, 200 nM each dinucleoside triphosphate (dNTP), and 0.25 μl of a 5 units/μl GoTaq DNA polymerase solution per sample, along with 200 nM each primer. After PCR, amplification was assessed by mixing 5 μl of each sample with 1 μl of 10,000× Sybr Safe dye run on a 1% agarose–Tris-borate-EDTA (agarose-TBE) gel. The replicate reaction mixtures were purified using Agencourt AMPure XP beads (Beckman Coulter, Inc., Pasadena, CA, USA) and quantified using the Qubit assay. Barcoded amplicons were sequenced using a 2 × 250-bp MiSeq Illumina format at the University of Illinois W. M. Keck Center for Comparative and Functional Genomics.

### Sequence processing.

Using mothur version 1.36.1 (46), barcodes and primers were first removed, leaving 8,849,002 sequences with an average length of 253 bp. The Silva rRNA sequence database (version 123) alignment template was used to align sequences to the 16S rRNA molecule. Sequences that were identified as Eukaryota, chloroplasts, mitochondria, or “unknown” were removed. This process reduced the total number of sequences to 8,446,696. Chimeras were detected using UCHIME (47) and removed, further reducing the number of sequences to 8,428,730. Nine samples each containing fewer than 10,000 sequences were then removed from the data set. Nonaligned versions of the sequences were then clustered using minimum entropy decomposition (MED), which applies Shannon entropy and uses the information-rich nucleotide positions across reads to iteratively partition large data sets while omitting stochastic variation (29), and it has been shown to produce patterns similar to the 97% sequence similarity criterion but with greater resolution of closely related species (48). The final data set contained 7,666,464 sequences categorized into 215 MED nodes. Sequences representing each MED were assigned a taxonomic group using a custom Silva rRNA gene sequence database (49) that was based on the nonredundant release version 123 and contained 484,763 additional sequences for Tenacibaculum and 1,989,583 additional sequences for Psychrobacter from the Silva version 123 redundant sequence database (http://www.whoi.edu/website/amy-apprill/SILVAdatabase). The custom database enhanced the placement of sequences affiliated with Tenacibaculum and Psychrobacter by 5.78% and 23.76%, respectively. The phylogeny of representative sequences of the MED nodes was manually examined against this same database using ARB with neighbor-joining phylogeny (50).

### Statistical analysis.

Using mothur, the alpha diversity of the skin microbial community was analyzed using nonrarified data (sequences ranged from 9,436 to 1,898,477, with a mean of 81,558 sequences per sample) by computing the number of observed MED nodes, Shannon's index (H′) (51), and inverse Simpson's index (52). Alpha diversity was compared according to sex, which was obtained using previously described methods (53), regional location (different bays, straits, etc.), and foraging season using the statistical package ‘stats’ in R. The beta diversity of the skin microbiome was assessed using Primer-E version 7 (PRIMER-E Ltd., Plymouth, UK) (54). Specifically, the relative abundances of the MED nodes were square root transformed and assembled into a distance matrix using Bray-Curtis similarity (55). These Bray-Curtis similarities were analyzed in the context of sex, regional location, and foraging season (early or late) using nonmetric multidimensional scaling (NMDS) ordination and hierarchical clustering analysis. PERMANOVA in Primer-E was used to test for significant differences in microbial community composition between sexes, among geographic locations, and between seasons with type III (partial) sum of squares with 999 permutations. Significance levels were confirmed using a Monte Carlo simulation. To avoid pseudoreplication, the averages of the replicate samples from the five resampled individuals were included in the analysis (except for the individual with replicate samples Mn_13_36a and Mn_13_37c, because these samples were collected in different bays).

Sea surface temperature (SST) at each sampling point was extracted from MODIS-Aqua monthly average SST satellite imagery at 9-km spatial scale using the freely available Marine Geospatial Ecology Toolkit (MGET) (56). SST was compared between early and late foraging seasons with an analysis of variance (ANOVA) from the statistical package in R.

### Defining core and common microbiome.

Defining core or persistent microbial groups in specific hosts or environment is typically done using a variety of approaches, with variations generally based upon a particular study's goals. Shade and Handelsman (31) suggest defining the persistence of a microbial group by the number of observations of operational taxonomic units that occur instead of using an arbitrary cutoff, as is often done in traditional ecology theory. In the present study, the most common MED nodes were affiliated with six main groups of bacteria, and within these groups, the MED nodes were found to be highly clustered, based on analysis in ARB using the custom SILVA data set described above. Because we used MED rather than a distinct sequence similarity threshold to define operational taxonomic units, and since data have not yet been produced to help understand if these closely related MED nodes are biologically or ecologically meaningful, we described the core microbiome using the most abundant group criteria. Groups of bacteria (generally genera) were characterized as core if they were present (i.e., detectable) in 93% or more of the skin samples and common if they were associated with 50 to 92% of the samples, and these levels of differentiation corresponded to natural divisions in the data. While the 100% membership criterion would have encapsulated most of our core MED nodes, dropping the criterion to 93% allowed the inclusion of marine mammal-specific sequences, which were widespread in our data set. Early and late foraging season skin samples were analyzed separately for the core and common microbiomes.

### Accession number(s).

Sequences were deposited at NCBI under BioProject no. PRJNA395930. Representative MED sequences are provided in File S2 in the supplemental material.

## Supplementary Material

Supplemental material
