# Supplementary material for: Temporal and Regional Variability in the Skin Microbiome of Humpback Whales along the Western Antarctic Peninsula
Source: Appl Environ Microbiol. 2018 Feb 14;84(5):e02574-17. doi: 10.1128/AEM.02574-17 (PMC5812929; doi:10.1128/AEM.02574-17)
Supplement: Supplemental material [file supp_84_5_e02574-17__index.html]

Supplemental material 

# Temporal and Regional Variability in the Skin Microbiome of Humpback Whales along the Western Antarctic Peninsula

## Supplemental material

- Supplemental file 1 -

  Box plot distributions (Fig. S1); nMDS analysis (Fig. S2); descriptions of the common microbiome for both early and late foraging season samples (Table S1); PERMANOVA results (Table S2).

  PDF, 412K
- Supplemental file 2 -

  Representative sequences and size of each MED node (Data Set S1).

  XLSX, 62K
