# Supplementary material for: Temporal and Regional Variability in the Skin Microbiome of Humpback Whales along the Western Antarctic Peninsula
Source: Appl Environ Microbiol. 2018 Feb 14;84(5):e02574-17. doi: 10.1128/AEM.02574-17 (PMC5812929; doi:10.1128/AEM.02574-17)
Supplement: Supplemental material [file AEM.02574-17_zam005188361s1.pdf]

**Supplementary Figures and Tables**

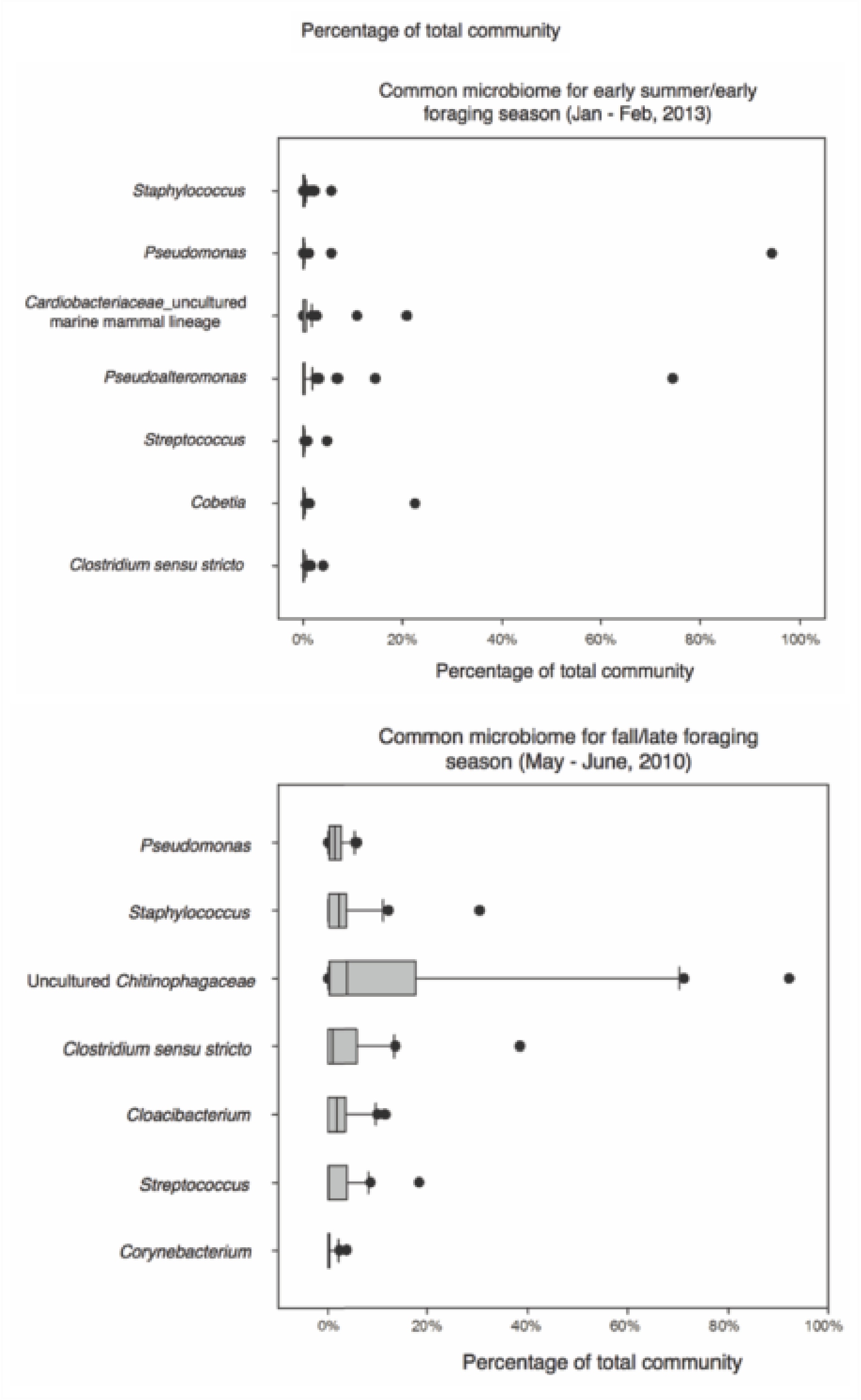


Figure S1. Boxplot distributions with average and standard deviation (sd) for the percent composition make up of each common genera for both early and late foraging season.

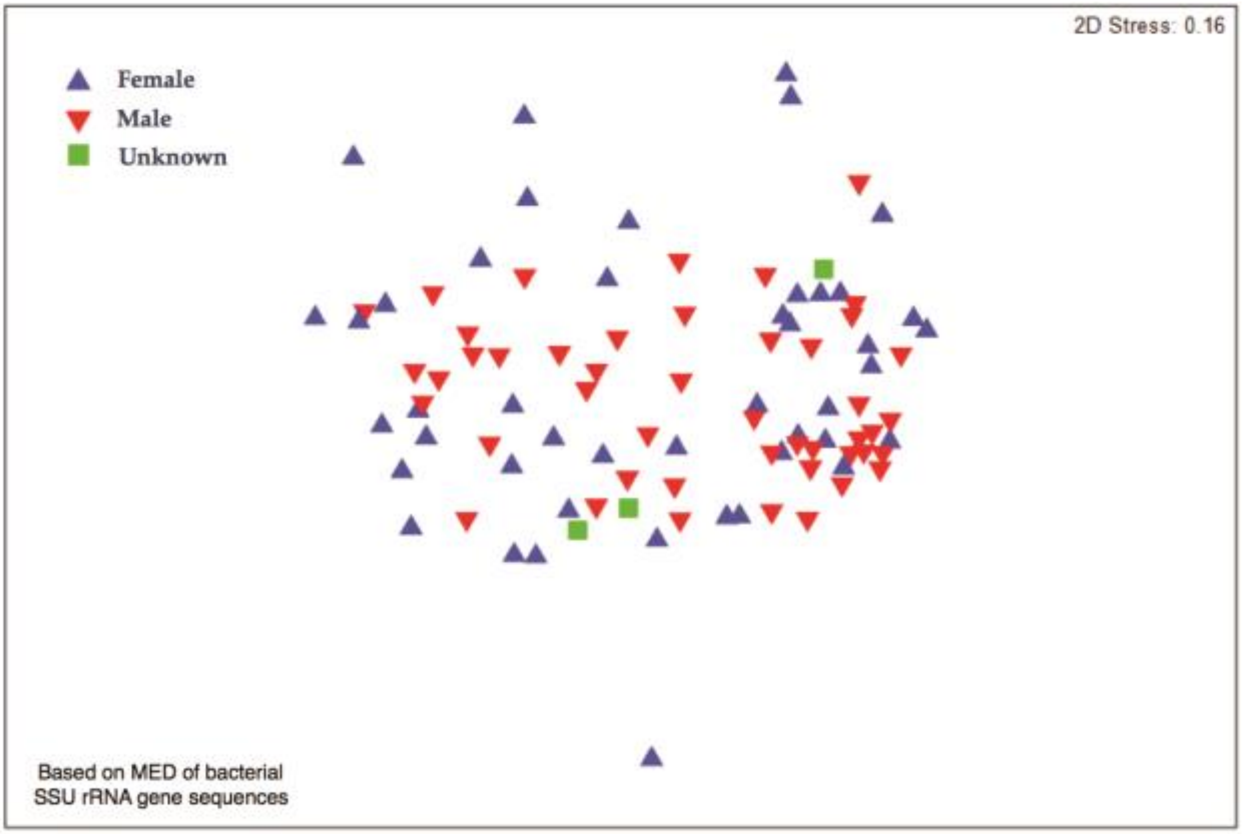


Figure S2. Nonmetric multidimensional scaling (nMDS) analysis based on skin-associated bacterial SSU rRNA genes of male and female humpback whales along the WAP (with three animals of unknown sex). PERMANOVA results showed no significant difference between sex and microbial composition ($p = 0.212$).

Table S1. Descriptions of the common microbiome for both early and late foraging season samples. Based on MED of bacterial SSU rRNA gene sequences.

| Taxonomic affiliation | Ratio found in samples (%) | # of OTUs in each Genus | Name of OTUs |
|---|---|---|---|
| Fall/late foraging season (May-June, 2010) | | | |
| *Pseudomonas* | 19:21 (90.5%) | 2 | 905, 3121 |
| *Staphylococcus* | 17:21 (81.0%) | 2 | 3183, 3186 |
| uncultured *Chitinophagaceae* | 16:21 (76.2%) | 1 | 1193 |
| *Clostridium* sensu stricto | 14:21 (66.7%) | 4 | 2685, 732, 37, 2686 |
| *Cloacibacterium* | 13:21 (61.9%) | 1 | 1198 |
| *Streptococcus* | 12:21 (57.1%) | 4 | 334, 333, 331, 335 |
| *Corynebacterium* | 11:21 (52.4%) | 1 | 105 |
| Early summer/early foraging season (Jan- Feb, 2013) | | | |
| *Staphylococcus* | 63:73 (86.3%) | 2 | 3183, 3186 |
| *Pseudomonas* | 59:73 (80.8%) | 2 | 905, 3121 |
| *Cardiobacteriaceae*, uncultured marine mammal associated bacterium | 56:73 (76.7%) | 8 | 3678, 817, 3059, 1094, 3058, 3065, 3061, 3687 |
| *Pseudoalteromonas* | 53:73 (72.6%) | 4 | 2783, 2813, 2812, 2784 |
| *Streptococcus* | 44:73 (60.3%) | 4 | 334, 333, 331, 335 |
| *Cobetia* | 39:73 (53.4%) | 1 | 1280 |
| *Clostridium* sensu stricto | 38:73 (52.1%) | 4 | 2685, 732, 37, 2686 |

Table S2. PERMANOVA results comparing skin microbiome between the eight locations along the WAP with ≥ 4 samples. Based on MED of bacterial SSU rRNA gene sequences.

| Groups | t | p | p (MC) |
|---|---|---|---|
| Andvord Bay vs. Charlotte Bay | 1.3041 | 0.094 | 0.124 |
| Andvord Bay vs. Flandres Bay | 1.7409 | 0.006** | 0.009** |
| Andvord Bay vs. Gerlache Strait | 2.0694 | 0.001*** | 0.001*** |
| Andvord Bay vs. Marguerite Bay | 1.887 | 0.007** | 0.006** |
| Andvord Bay vs. Palmer Deep Canyon | 1.9642 | 0.006** | 0.008** |
| Andvord Bay vs. Palmer Station | 1.3794 | 0.104 | 0.102 |
| Andvord Bay vs. Wilhemina Bay | 0.9923 | 0.389 | 0.391 |
| Charlotte Bay vs. Flandres Bay | 1.0532 | 0.275 | 0.373 |
| Charlotte Bay vs. Gerlache Strait | 1.2097 | 0.14 | 0.164 |
| Charlotte Bay vs. Marguerite Bay | 1.5344 | 0.021* | 0.051 |
| Charlotte Bay vs. Palmer Deep Canyon | 1.751 | 0.013* | 0.026* |
| Charlotte Bay vs. Palmer Station | 1.3334 | 0.104 | 0.13 |
| Charlotte Bay vs. Wilhemina Bay | 0.91697 | 0.463 | 0.47 |
| Flandres Bay vs. Gerlache Strait | 0.95464 | 0.501 | 0.488 |
| Flandres Bay vs. Marguerite Bay | 2.0219 | 0.002** | 0.006** |
| Flandres Bay vs. Palmer Deep Canyon | 2.0113 | 0.003** | 0.005** |
| Flandres Bay vs. Palmer Station | 1.6167 | 0.017* | 0.039* |
| Flandres Bay vs. Wilhemina Bay | 1.3155 | 0.084 | 0.113 |
| Gerlache Strait vs. Marguerite Bay | 2.1596 | 0.002** | 0.001*** |
| Gerlache Strait vs. Palmer Deep Canyon | 1.9627 | 0.004** | 0.004** |
| Gerlache Strait vs. Palmer Station | 1.5913 | 0.026* | 0.029* |
| Gerlache Strait vs. Wilhemina Bay | 1.6264 | 0.019* | 0.031* |
| Marguerite Bay vs. Palmer Deep Canyon | 0.86522 | 0.619 | 0.618 |
| Marguerite Bay vs. Palmer Station | 0.78181 | 0.782 | 0.719 |
| Marguerite Bay vs. Wilhemina Bay | 1.3948 | 0.089 | 0.082 |
| Palmer Deep Canyon vs. Palmer Station | 0.95054 | 0.488 | 0.474 |
| Palmer Deep Canyon vs. Wilhemina Bay | 1.4711 | 0.063 | 0.072 |
| Palmer Station vs. Wilhemina Bay | 1.0201 | 0.368 | 0.36 |
| *p≤0.05, **p≤0.01, ***p≤0.001 | | | |
